# Rapid initial decline in BCR-ABL1 is associated with superior responses to second-line nilotinib in patients with chronic-phase chronic myeloid leukemia

**DOI:** 10.1186/1471-2407-13-173

**Published:** 2013-04-02

**Authors:** Andrew M Stein, Giovanni Martinelli, Timothy P Hughes, Martin C Müller, Lan Beppu, Enrico Gottardi, Susan Branford, Simona Soverini, Richard C Woodman, Andreas Hochhaus, Dong-Wook Kim, Giuseppe Saglio, Jerald P Radich

**Affiliations:** 1 Novartis Institutes for BioMedical Research, Inc., 45 Sidney St, Cambridge, MA 02139, USA; 2 University of Bologna, Department of Hematology and Oncological Sciences “L. e A. Seràgnoli, Bologna, Italy; 3 Centre for Cancer Biology/SA Pathology, Royal Adelaide Hospital, Adelaide, Australia; 4 Medizinische Fakultät Mannheim der Universität Heidelberg, Mannheim, Germany; 5 Clinical Research Division, Fred Hutchinson Cancer Research Center, Seattle, WA, USA; 6 University of Turin, Orbassano, Italy; 7 Novartis Pharmaceuticals Corporation, East Hanover, NJ, USA; 8 Universitätsklinikum Jena, Jena, Germany; 9 Seoul St. Mary’s Hospital, The Catholic University of Korea, Seoul, Korea

**Keywords:** Chronic myeloid leukemia, Nilotinib, Mathematical modeling, BCR-ABL, Molecular response

## Abstract

**Background:**

We evaluated BCR-ABL1 kinetics in patients treated with nilotinib and analyzed whether a dynamic model of changes in BCR-ABL1 levels over time could be used to predict long-term responses.

**Methods:**

Patients from the nilotinib registration trial (CAMN107A2101; registered at http://www.clinicaltrials.gov as NCT00109707) who had imatinib-resistant or -intolerant Philadelphia chromosome–positive (Ph+) chronic myeloid leukemia (CML) in chronic phase (CP) or accelerated phase with BCR-ABL1 > 10% (on the international scale [IS]) at baseline and, in the first 6 months, had at least three BCR-ABL1 transcript measurements and an average daily dose of at least 720 mg were included in this analysis (N = 123).

**Results:**

More than half of patients (65/123; 53%) had a slow monophasic response and the remainder (58/123; 47%) had a biphasic response, in which patients had a rapid initial decrease in BCR-ABL1 transcripts followed by a more gradual response. The biphasic response type strongly correlated with improved event-free survival (EFS). Data in the first 6 months of follow-up were sufficient to predict EFS at 24 months.

**Conclusions:**

Unlike newly diagnosed patients with Ph+ CML-CP—in whom the majority had a biphasic response—approximately half of patients with imatinib-resistant or -intolerant CML had a slower, monophasic response. Second-line patients who did have a biphasic response had an EFS outlook similar to that of newly diagnosed patients treated with imatinib. Our model was comparable to using BCR-ABL1 (IS) ≤ 10% at 6 months as a threshold for predicting EFS.

## Background

Chronic myeloid leukemia (CML) is a clonal myeloproliferative disorder characterized by the expansion of hematopoietic cells carrying the oncogenic *BCR-ABL1* fusion gene. The *BCR-ABL1* fusion gene is formed by a reciprocal translocation of a fragment of the Abelson gene (*ABL1*), situated at the breakpoint on chromosome 9, with a fragment of the breakpoint cluster region (*BCR*) gene on chromosome 22. This translocation forms the Philadelphia chromosome (Ph), which encodes the constitutively active BCR-ABL1 protein tyrosine kinase [1,2]. Deregulated phosphorylation of protein tyrosine residues by this constitutively active tyrosine kinase leads to activation of downstream effectors that enable growth factor–independent proliferation and neoplastic transformation in the transformed hematopoietic cells [3].

Imatinib is a BCR-ABL1 tyrosine kinase inhibitor (TKI) that demonstrated superior response rates and improved tolerability over interferon-α plus cytarabine in the phase III open-label International Randomized Study of Interferon and STI571 (IRIS) trial [4]. Despite the success of imatinib, a significant proportion of patients discontinue therapy (34% in the IRIS trial) [5] for reasons that include intolerance or resistance to the drug.

Nilotinib was rationally designed after the discovery of resistance to imatinib in early clinical trials [6-8] and laboratory evidence showing that point mutations in the BCR-ABL1 kinase domain mutation were the most common cause of such resistance [9]. A more potent BCR-ABL1 kinase inhibitor was hypothesized to reduce the reservoir of leukemic cells in patients, thereby impeding the emergence of drug resistance. Nilotinib is approved in more than 60 countries worldwide for the treatment of newly diagnosed patients with Ph+ CML in the chronic phase (CP) and in patients with Ph+ CML-CP and in the accelerated phase (AP) who have failed prior therapy, including imatinib. Approval as second-line treatment was based on results from a phase II open-label registration study [10] that showed durable responses and overall survival of 87% with 24 months of follow-up [11].

Normalization of blood counts, as measured by hematologic responses; reduction and elimination of the Ph chromosome, as measured by cytogenetic monitoring; and reduction and elimination of *BCR-ABL1* gene expression, as measured by real-time quantitative polymerase chain reaction (RQ-PCR) are important measures of treatment success. Before the routine use of BCR-ABL1 TKIs, the evaluation of hematologic and cytogenetic responses was sufficient to determine treatment efficacy, as improvement in these outcomes was the limit of response in most patients. However, with more potent BCR-ABL1–targeting therapies, deeper responses are achieved in most patients, necessitating more sensitive methods of disease detection.

An abundance of literature explores the use of molecular response to predict long-term outcome [12-18]. A common approach is the landmark analysis, whereby a particular threshold (e.g., BCR-ABL1 ratio ≤ 0.1% on the international scale [IS]) and time (e.g., 3 months) are chosen, and longer-term outcomes (e.g., progression-free survival or event-free survival [EFS] at 2 years) are evaluated according to whether patients attained the threshold at the landmark time point [18-23]. While this approach is reasonable in controlled clinical trials, challenges may arise in practice, including missing samples or those inconsistent with prior or subsequent samples. For example, a patient may not have a BCR-ABL1 assessment at month 6 but may have assessments at months 4 and 8. Or, a patient may achieve major molecular response (MMR) at month 6, lose MMR at month 12, and then regain MMR at month 18. Rather than assessing a patient based on his or her response at a single time point, using all of the information from a patient’s full clinical history may provide a more robust method for classifying patient response.

A mathematical model is a natural tool for integrating information from the full BCR-ABL1 transcript dynamic time course. Several groups have established such models for predicting BCR-ABL1 response in patients with CML [13,15,18]. We previously presented an approach using standard pharmacokinetic/pharmacodynamic methods to model the clinical data of newly diagnosed patients treated with imatinib in the IRIS trial [18]. Here, we applied this model to patients with CML who received imatinib treatment that had failed because of resistance or intolerance and who were subsequently treated with nilotinib in the registration study. The objectives of this analysis were to (1) determine whether BCR-ABL1 kinetics in patients treated with nilotinib were similar to those in patients treated with imatinib; (2) explore whether a model using BCR-ABL1 as a continuous measurement could potentially be used to predict long-term response based on short-term data; and (3) compare such methods with simpler methods.

## Methods

### Patients and samples

The nilotinib registration trial (CAMN107A2101; registered at http://www.clinicaltrials.gov as NCT00109707) was an open-label, multicenter, single-arm phase II study of nilotinib (400 mg twice daily) in patients with imatinib-resistant or -intolerant Ph+ CML-CP (N = 321; 226 imatinib-resistant, 95 imatinib-intolerant) with a median exposure to nilotinib of 561 days [10,11]. This study was conducted in accordance with the Declaration of Helsinki, and the protocol was reviewed by the ethics committee or institutional review board at each participating institution. All patients gave written informed consent.

RQ-PCR measurements of BCR-ABL1 were taken monthly during the first 3 months of the trial and once every 3 months thereafter, as described previously [24]. For this analysis, patients were required to have at least three PCR measurements, an average daily dose of at least 720 mg (90% of the target dose) in the first 6 months of the study, and BCR-ABL1 (IS) > 10% at baseline (n = 123).

BCR-ABL1 (IS) ≤ 10% can be measured effectively [25]. The upper limit of 10% is determined by two sources of nonlinearity in the BCR-ABL1 ratio estimation: (1) depending on the primer design for the *ABL1* control gene, both *ABL1* and *BCR-ABL1* are amplified when the BCR-ABL1 levels are high [26]; and (2) healthy cells have two copies of the *BCR* and *ABL1* control genes, while leukemic cells have one control gene and one *BCR-ABL1* gene. There is no simple formula that can be applied to remove this nonlinearity because expression rates of the control genes may be different at the time of diagnosis and at the time that a patient achieves a good response. Thus, while measurements above 10% are included in this analysis so that we can describe the full range of BCR-ABL1 dynamics for all patients, quantitative values of the parameters governing the dynamics at the start of treatment (A, α, μ; see model below) should be considered qualitatively.

### Model selection and parameter fitting

The time course of BCR-ABL1 transcript levels was modeled using nonlinear mixed effects (NLME) methodology. This is an accepted approach for deriving population models from clinical data [27,28]. We used maximum likelihood methods, as implemented in Monolix (http://www.lixoft.com/wp-content/resources/docs/UsersGuide.pdf) NLME software in MATLAB (The MathWorks, Inc., Natick, MA), to fit the following between-patient mixture model to the log_10_ of the BCR-ABL1 ratios measured at time t, R(t), where treatment begins at t = 0:

$$R\left(t\right)=\{\begin{array}{l} log_{10}\left(Ae^{\mathrm{\mu t}}\right)+\varepsilon \;\mathrm{Slow}\;\mathrm{BCR}-\mathrm{ABL}\\ \;\mathrm{respons}e\;\mathrm{with}\;\mathrm{probability}\;p\\ log_{10}\left(Ae^{\mathrm{\alpha t}}+Be^{\mathrm{\beta t}}\right)+\varepsilon \;\mathrm{Fast}\;\mathrm{BCR}-\mathrm{ABL}\\ \;\mathrm{response}\;\mathrm{with}\;\mathrm{probability}\;1-p \end{array}$$

The model represents two typical profiles: (1) slow responders with a monophasic decline, who typically had little if any decline in BCR-ABL1 (IS)% (at rate μ), and (2) fast responders with a biphasic decline, in whom a rapid initial decrease in the BCR-ABL1 (IS)% (at rate α) was followed by a more gradual response (at rate β) that could be either increasing or decreasing. A similar version of the above model was used to fit patients from the IRIS study [18], although we excluded the triphasic mixture population due to limited follow-up time (2 years) in the present study. The constant terms (A, B) were restricted to be greater than zero because BCR-ABL1 (IS)% is always positive. The rate constants (μ, α, β) have units of 1/year. Before treatment starts, the log_10_ of the BCR-ABL1 (IS)% is assumed to be log(A) or log(A + B) with additive measurement error. The model has an additive residual error term (ε) in log space that describes variability in the measurement due to assay and interoccasion variability and model error. All mono- or biexponential parameters (A, B, α, β, μ) were treated as mixed effects with both a fixed component that represents the typical value for the population and a random component that represents the interpatient variability. The mixture probability p was a fixed effect. The constant terms (A, B) were chosen from a log-normal distribution because BCR-ABL1 (IS)% is always greater than zero (e.g., A = θ_Α_*exp(η_Α_), where η_Α_ is normally distributed with mean zero and variance ω_A_). Similarly, both α and μ are always less than or equal to zero and so are chosen from a log-normal distribution with a negative fixed-effect term (e.g., θ_α_ < 0). Thus, we have α = θ_α_*exp(η_α_). Finally, we chose β from a normal distribution, allowing it to be either negative or positive (i.e., β = θ_β_ + η_β_).

We used 0.0032% (a 4.5-log_10_ decrease in the BCR-ABL1 [IS]%) as the minimal level of detection of the PCR assay for all laboratories and treated these measurements as left-censored data. BCR-ABL1 transcript levels below 0.0032% were established as “undetectable” in the IRIS trial [29], and BCR-ABL (IS) ≤ 0.0032% has subsequently been defined as molecular remission at 4.5 logs (MR^4.5^) [30,31]. Rather than remove all measurements below the limit of quantitation or fix them at a certain value, we used the M3 maximum-likelihood approach [32], which treated any measurement falling below the limit of detection as having an upper boundary of 0.0032% to make optimal use of all measurements.

The maximum-likelihood approach yields information about both the population and each individual patient. For the population, the typical model parameters (A, B, μ, α, β, p) and the interpatient variability (of A, B, μ, α, β) were estimated. In addition, for each individual, the most likely set of parameters that describe that individual was estimated, along with the patient’s BCR-ABL1 response classification (slow vs. fast). In particular, each patient was categorized as a fast or slow responder based on the best description of that patient as calculated by the maximum-likelihood method. For the biphasic patients, we also computed the transition time at which the patient transitioned from the α to the β phase of the decline. We defined the transition time as the point of maximum curvature of the fitted BCR-ABL1 transcript profile, where curvature was defined in the differential geometrical sense as κ(t) = *|R”(t)|/(1 + R’(t)*^*2*^*)*^*3/2*^.

### Response prediction and model comparison

We then tested the ability of the model to predict the event status of individual patients at year 2 from data in the first 1, 3, 6, 9, 12, and 18 months. EFS was defined as the time between randomization and any of the following events on treatment: death due to any cause, progression to AP or blast crisis, loss of complete hematologic response, or loss of major cytogenetic response. For each landmark time, patients who progressed or died before that time were excluded from the analysis.

Categorization was performed by fixing the average model parameters for the population (A, B, μ, α, β, p) and then using the data from baseline to the landmark time of interest to estimate the individual parameters and response classification (fast vs. slow responder) for each patient. When fewer than 6 months of data were used, the long-term response parameters B and β were not estimable, in which case the model naturally assumed the population estimates for each patient. Because the purpose of this analysis was to categorize each patient as a fast or slow responder, the estimation of the long-term response was not essential.

Finally, we compared the models’ ability to predict event status at 2 years based on a single BCR-ABL1 transcript measurement at 3 or 6 months, respectively. We predicted each patient’s event status by whether the BCR-ABL1 levels were above or below a particular threshold at a particular landmark time. The thresholds used were BCR-ABL1 (IS) > 10%, > 1%, and > 0.1%, and the landmark times were 3 and 6 months. The models were compared according to the percentage of patients who were correctly classified, the positive predictive value (PPV; proportion of nonprogressing patients correctly classified as nonprogressing), and the negative predictive value (NPV; proportion of progressing patients correctly classified as progressing). In this case, we restricted the analysis to the 76 patients who did not progress by month 6 and whose event status by year 2 was known.

## Results

**Figure 1:**
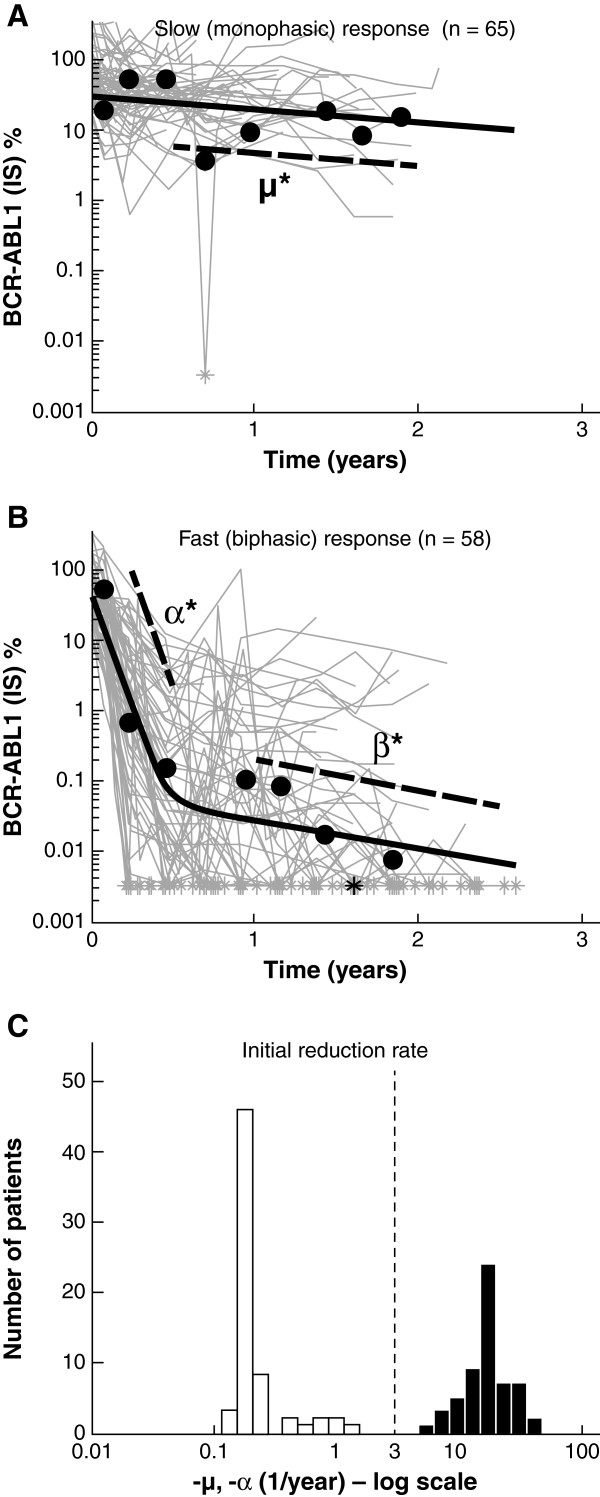
**BCR-ABL1 (international scale [IS])% for all 123 patients in this analysis, divided into two groups, with an example patient shown in black within each group: (A) slow monophasic responders (n = 65) with μ = −0.4/year for the example patient and (B) fast biphasic responders (n = 58) with α = −15.1/year and β = −0.9/year for the example patient.** The μ*, α*, β* parameters are shown for the example patient, where μ* = μlog_10_e, α* = αlog_10_e, and β* = βlog_10_e. The asterisks (*) indicate when a BCR-ABL1 (IS)% measurement fell below 0.0032% (= 100 × 10^-4.5^), the approximate limit of quantitation of the assay for all laboratories in the International Randomized Study of Interferon and STI571 trial; (**C**) histogram of the initial reduction rates μ and α. Note that there is no overlap between the two populations and that the vertical dashed line at −3/year demonstrates where the separation occurs.

**Figure 2:**
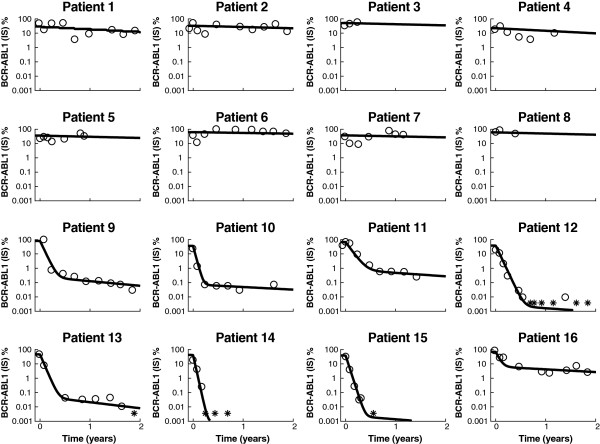
**Individual fits to 16 patients. Patients 1 to 8 are slow monophasic, and patients 9 to 16 are fast biphasic.** The asterisk (*) represents measurements below the limit of quantification of the assay.

Of the total 123 patients analyzed, 65 (53%) were categorized as slow monophasic responders and 58 (47%) as fast biphasic responders (Figures 1A and 1B). Individual fits to 16 representative patients are shown in Figure 2. Note that some patients (patients 14 and 15) obtained an undetectable level of transcripts before transitioning to the β phase. The model accounts for these patients by treating them as biphasic responders with a β phase below the limit of quantification of the assay, although the true dynamics below 0.0032% BCR-ABL1 are unknown.

The parameter p was estimated accordingly to be 51% ± 10%, differing significantly from IRIS, where the probability of a monophasic response was only 14%. However, the other model parameters were all comparable, with typical values of: θ_A,IRIS_ = 34%, θ_A,2101_ = 45%; θ_B,IRIS_ = 0.09%, θ_B,2101_ = 0.05%; θ_μ,IRIS_ = −0.1/year, θ_μ,2101_ = −0.2/year; θ_α,IRIS_ = −14/year, θ_α,2101_ = −17/year; θ_β,IRIS_ = −0.5/year, θ_β,2101_ = −0.6/year [18]. In addition, the variances on the parameters from 2101 were ω_A_ = 0.4, ω_B_ = 8.8, ω_μ_ = 1.4, ω_α_ = 0.3, ω_β_ = 0.3. The large variability in B indicates that the β-phase transition for the biphasic patients can occur over a large range of BCR-ABL1 values, from below 0.0032% (undetectable) to 7%. The initial response rate—which was equal to μ for the slow responders and α for the fast responders—had a bimodal distribution, with a threshold of approximately −2 to −6/year separating the two distributions (Figure 1C).

A fast biphasic response, according to the model, was associated with the best hematologic, cytogenetic, and molecular responses by 24 months, and patients classified as fast responders were more likely to be considered optimal responders according to European LeukemiaNet criteria [33] (Table 1). Furthermore, slow monophasic responders were more likely to be imatinib-resistant (as opposed to imatinib-intolerant) and have nilotinib-insensitive mutations (half maximal inhibitory concentration [IC_50_] > 150 nm; Y253H, E255V/K, F359V/C) [24] in BCR-ABL1 at baseline (Table 2).

**Table 1:** Patient response by model response type

		**Best response achieved over 24 months, n (%)**			**ELN response at 12 months, n (%)**			
**Response type**	**All patients, n**	**CHR**	**CCyR**	**MMR**	**Optimal**	**Suboptimal**	**Warning or failure**	**Not assessable**
Slow (monophasic)	65	47 (72.3)	5 (7.7)	1 (1.5)	2 (3.1)	2 (3.1)	39 (60.0)	22 (33.8)
Fast (biphasic)	58	57 (98.3)	51 (87.9)	41 (70.7)	40 (69.0)	7 (12.1)	4 (6.9)	7 (12.1)

*Abbreviations*: CCyR = complete cytogenetic response; CHR = complete hematologic response; ELN = European LeukemiaNet; MMR = major molecular response.

**Table 2:** Patient baseline characteristics by model response type

**Response type**	**All patients, n**	Sensitive baseline mutation,^* ^**n (%)**	**Insensitive baseline mutation,**^**† **^**n (%)**	**Imatinib-resistant, n (%)**	**Imatinib-intolerant, n (%)**
Slow (monophasic)	65	21 (32.3)	18 (27.7)	58 (89.2)	7 (10.8)
Fast (biphasic)	58	18 (31.0)	2 (3.4)	42 (72.4)	16 (27.6)

^*^ Half maximal inhibitory concentration (IC_50_) ≤ 150 nm.

^**†**^ IC_50_ > 150 nm; Y253H, E255V/K, F359V/C.

A small number of patients achieved a fast response without a detectable β phase. These patients had small estimated B values, indicating that the transition from the α to the β phase occurred below MR^4.5^. The true BCR-ABL1 dynamics below MR^4.5^ cannot be accurately determined at present.

**Figure 3:**
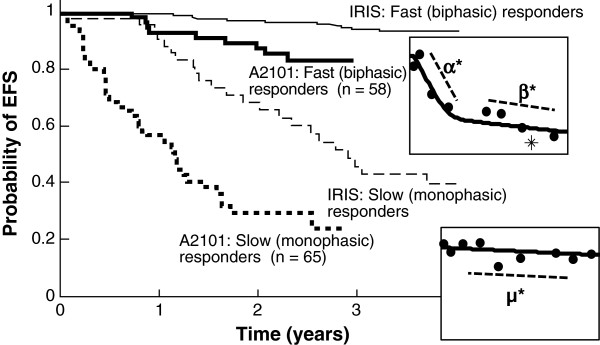
**Event-free survival (EFS) according to patient group.** The Kaplan-Meier plot of the imatinib arm of the International Randomized Study of Interferon and STI571 (IRIS) trial for frontline patients with chronic myeloid leukemia is also shown, to illustrate that biphasic responders do approximately as well as patients receiving frontline imatinib treatment.

The EFS outcomes of the two responder groups are shown in Figure 3, overlaid with the EFS for newly diagnosed patients with CML in the IRIS trial. Fast responders on second-line nilotinib had higher EFS rates than did slow responders and had a comparable outlook to that of a typical patient on frontline imatinib.

**Figure 4:**
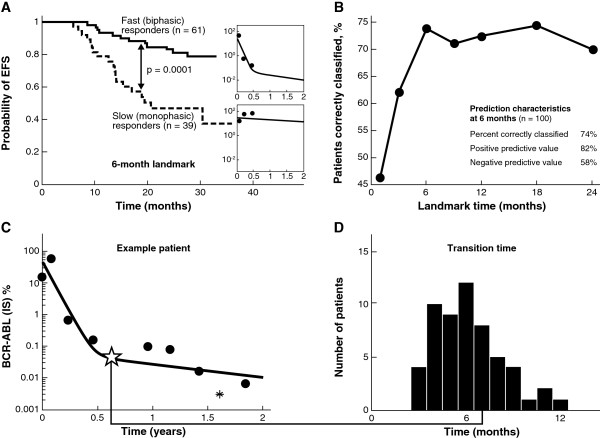
**(A) Event-free survival (EFS) at 2 years based on patient classification using only the first 6 months of data.** The difference between the EFS curves was found to be statistically significant at p = 0.0001 by a log-rank test; (**B**) the percentage of patients correctly classified as having an event at year 2 as a function of the amount of data used to classify each patient; (**C**) example patient: the ✩ indicates the transition point, defined as the point of maximum curvature where fast responders transition from the α to the β phase; the * corresponds to measurements below the limit of quantification of the polymerase chain reaction assay, assumed to be 0.0032% for all labs; (**D**) histogram of transition times between the α and β phases. *Abbreviation*: IS = international scale.

Assuming the same average population parameters with different landmark times, we assessed the ability of the model to predict EFS at 2 years. At a landmark time of 6 months, the model showed a statistically significant difference in EFS between patients classified as slow (monophasic) and fast (biphasic) responders (Figure 4A). We changed the follow-up time in the model from 1 month to 18 months and found that the classification improved up to month 6, but after that there was no improvement in the percentage of patients correctly classified as having progressed (Figure 4B). The median time to transition from the α to the β phase for biphasic patients was 6.1 months, with 66% of these patients having a transition point between months 4 and 8 (Figures 4C and 4D). The mean BCR-ABL1 ratio at the time of the transition was 0.035%.

Finally, we compared model classification to other predictive measures of patient response, in particular, the molecular response at 3 and 6 months (Table 3). We found that the model classification had the highest percentage of correctly classified patients as well as the highest NPV. However, the improvement shown in the model-based classification over a simpler classification method—one categorizing patients based on whether their BCR-ABL1 (IS) was above or below 10% at 6 months—was not substantial (2.6%). We also found that the model using 6 months of follow-up was more accurate at predicting EFS at 2 years than the model using 3 months of follow-up.

**Table 3:** **Comparison of different categorization methods for predicting event-free survival at 2 years in patients alive and event free at 6 months (n** ***=*** **76)**

**Follow-up time for subpopulation predicted to progress**	**N (above, below) threshold or (slow, fast) response**	**Correctly classified,%**	**Positive predictive value,%**	**Negative predictive value,%**
3 months				
> 10% BCR-ABL	32, 44	67.1	81.8	46.9
> 1% BCR-ABL	51, 25	52.6	84.0	37.3
> 0.1% BCR-ABL (MMR)	63, 13	44.7	92.3^*^	34.9
6 months				
> 10% BCR-ABL	26, 50	75.0	84.0	57.7
> 1% BCR-ABL	38, 38	64.5	84.2	44.7
> 0.1% BCR-ABL (MMR)	50, 26	56.6	88.5	40.0
Slow (monophasic) responders	27, 49	77.6^*^	86.0	61.5^*^

*Abbreviation*: MMR = major molecular response.

^*^ The number in bold indicates the highest score for that column.

## Discussion

A model-based analysis was previously developed to classify patients from the imatinib arm of the IRIS trial into three main categories: (1) slow monophasic patients who either do not respond to therapy or exhibit a very gradual BCR-ABL1 decline; (2) fast biphasic patients with an initial rapid decline in BCR-ABL1 during the first 6 months of therapy, followed by a more gradual decline; and (3) fast triphasic patients who follow the biphasic trend but relapse 2 to 4 years into treatment, exhibiting a rapid rise in BCR-ABL1 [18]. Because second-line patients treated with nilotinib in the registration trial have only been followed for 2 to 3 years thus far, the triphasic patients have been difficult to detect. Thus, we applied a scaled-down version of the model that categorized patients as either slow or fast BCR-ABL1 responders.

Patients were nearly evenly divided between the slow monophasic population (65/123; 53%) and the biphasic population (58/123; 47%). This was in contrast to the IRIS trial [18], where the vast majority of patients exhibited a biphasic response. However, the biphasic patients in the nilotinib registration trial had a similar EFS outlook to that of newly diagnosed biphasic patients in the imatinib arm of the IRIS trial, and the model parameters describing the monophasic and biphasic parameters were similar for IRIS and the nilotinib registration trial. This could be because the resistance to imatinib was overwhelmed by the more potent BCR-ABL inhibition by nilotinib and/or because "resistance" was actually intolerance or nonadherence, whereby patients did not take imatinib daily. Thus, some of the nilotinib biphasic responders may have actually been patients intolerant to or nonadherent with imatinib who might have been responders had they remained adherent to their medication.

The biphasic response type strongly correlated with improved EFS, as in IRIS. In addition, for this patient subpopulation, a 6-month follow-up time was optimal for predicting EFS at 24 months. This coincides with the observation that the transition time from the α to the β phase in biphasic patients typically occurs between months 4 and 8. Additional measurements at later times were of limited value for prediction based on model categorization. The median transition time at 6 months, at a median transition depth of 0.035% BCR-ABL1 (IS), also provides insight into why using a single measurement at 6 months outperforms using a measurement at 3 months. This level of response—nearly a 4-log reduction in BCR-ABL1 from standardized baseline—is a deeper response than MMR (BCR-ABL1 [IS] ≤ 0.1%), and achieving such a response has been associated with excellent long-term outcomes [22,23,34].

These findings are consistent with other studies showing that early molecular responses were associated with superior EFS and overall survival [22,35-37]. These landmark analyses, most in frontline patients treated with imatinib or imatinib-based combinations, typically focused on responses at 3 months as a predictor of later responses. Recent data from patients who received second-generation TKIs as second-line treatment at the Hammersmith Hospital in London revealed that molecular responses at 3 months were predictive of overall survival [38]. Another landmark analysis of imatinib-resistant or -intolerant patients enrolled in the nilotinib registration trial showed that molecular responses at 3 months predicted EFS at 24 months [21], but did not compare the utility of 3- versus 6-month BCR-ABL levels. A recent evaluation of frontline dasatinib suggested that the predictive value of the 3-month landmark could be improved with drug-specific BCR-ABL cutoffs [39].

We note that it may seem counterintuitive to use this model to categorize a patient as a fast biphasic responder when only 3 to 6 months of data is available, and the β phase is not yet observable. In using the biphasic model even in the presence of limited information, we are making use of prior information from both IRIS as well as this study, where almost all fast responders ultimately have a β phase. Furthermore, in some cases, the β phase does occur before month 6 (Figure 4D), so accounting for this second slope may be important for these patients.

Because the definition of the transition point as the point of maximum curvature was somewhat complex, we initially tested simpler definitions for the transition point, such as the point of maximum second derivative (*R″(t)*), or the point where *Ae*^*αt*^ *= Be*^*βt*^. We found that the point of maximum curvature definition most consistently agreed with our estimation of where the transition point should lie based on clinical experience.

The model used in this study was not superior at predicting the longer-term event status of individual patients versus using BCR-ABL1 (IS) above or below 10% at 6 months. Furthermore, the NPV of all the classifiers in the model was relatively low (maximum of 62%). This may be due in part to 2 years of follow-up being too short and that, over longer times, the monophasic patients may continue to be more likely to progress than the biphasic patients. The NPVs and PPVs depend on the prevalence of second-line patients who do not respond well to nilotinib therapy; this prevalence may be lower in a controlled trial than in the general population. Nevertheless, PPV and NPV are preliminary tools for comparing the predictive power of different methodologies. Our modeling approach represents one of many classification schemes that can be used for dividing a population into two groups. In the future, we could consider exploring other classification approaches, such as neural networks or support vector machines.

Key benefits of the modeling approach (classification of a second-line patient as either a fast or slow responder) are that (1) the model helps to identify 6 months as an ideal follow-up time; and (2) the model provides a natural means for incorporating all measurements and treating the BCR-ABL1 dynamics as a continuous variable up to the time at which classification takes place. In the clinic, samples may be missed or multiple conflicting measurements may be obtained in the same time period. In these instances, a simple algorithm may not be able to adequately determine patient response based on one data point at a particular time. The model, however, provides a natural technique for integrating missing data and multiple samples at the same time, and produces a dichotomous classification associated with EFS. While we required patients to have at least three data points in the first 6 months, the NLME approach used here can work with fewer data points such that a patient with only one assessment at month 6 could be classified as a fast responder if he or she has achieved BCR-ABL1 (IS) ≤ 0.1% (at least MMR) or a slow responder if his or her BCR-ABL1 levels are above 10%. In the future, it will be useful to investigate how the model performance changes depending on the amount of data collected.

A related modeling approach that also treated BCR-ABL1 response as a continuous parameter was recently reported [40]. In this model, BCR-ABL1 doubling time was used to characterize relapse rate. Patients entering blast crisis had similar BCR-ABL1 doubling times to patients discontinuing therapy, whereas patients with BCR-ABL–resistant mutations who remained in CP had a longer time to relapse. These differences in the velocity of leukemic cell growth were not always evident by the BCR-ABL1 fold rise, which was dependent on the time between measurements.

The nilotinib registration trial demonstrated high rates of major cytogenetic response (59%) and complete cytogenetic response (CCyR; 44%) [11]. Most patients who achieved CCyR also achieved MMR (56%), and cytogenetic responses were durable, with 84% of patients who achieved CCyR maintaining response at 24 months. The overall survival at 24 months was 87%. Here, we showed that the EFS rates of fast responders to nilotinib were comparable to those observed with newly diagnosed patients treated with imatinib, and that BCR-ABL1 dynamics in the first 6 months of therapy were useful in predicting such responses.

This work represents an exploratory step in using a model to measure response based on BCR-ABL1 as a continuous variable and transform it into a dichotomous classification associated with EFS. Because the patient subpopulation in this study was highly heterogeneous and the subpopulation used to compare different predictors was small (n = 76), more testing and validation will be necessary before this approach can be directly applied in the clinical setting.

## Conclusions

Landmark analyses, in which longer-term outcomes are analyzed according to achievement of molecular milestones at early time points (3 and 6 months), have become an increasingly common means of evaluating responses to both front- and second-line TKI therapies used for the treatment of CML. However, this emphasis on achieving specific molecular targets may be challenging in routine clinical practice, where samples are frequently missing. Here, we described a mathematical model that utilizes the full BCR-ABL1 transcript profile to predict longer-term responses among patients treated with nilotinib after imatinib failure. Evaluating BCR-ABL1 transcript dynamics over time in an individual patient may provide a more robust and practical method for classifying patient response.

## Abbreviations

CML: Chronic myeloid leukemia; ABL1: Abelson gene; BCR: Breakpoint cluster region; Ph: Philadelphia chromosome; IRIS: International Randomized Study of Interferon and STI571; CP: Chronic phase; AP: Accelerated phase; RQ-PCR: Real-time quantitative polymerase chain reaction; IS: International scale; EFS: Event-free survival; MMR: Major molecular response; NLME: Nonlinear mixed effects; MR4.5: Molecular remission at 4.5 logs; PPV: Positive predictive value; NPV: Negative predictive value; IC50: Half maximal inhibitory concentration; CCyR: Complete cytogenetic response.

## Competing interests

Dr Stein is an employee of Novartis. Dr Martinelli acted as a consultant for Novartis, Bristol-Myers Squibb, Merck Sharp & Dohme, Pfizer, and Genzyme, and received honoraria from Novartis and Bristol-Myers Squibb. Dr Hughes acted as a consultant for and received honoraria and research funding from Novartis and Bristol-Myers Squibb. Dr Müller acted as a consultant for and received honoraria from Novartis and Bristol-Myers Squibb and received research funding from Novartis. Dr Beppu and Dr Gottardi have nothing to disclose. Dr Branford acted as a consultant for and received research funding from Novartis and received honoraria from Novartis and Bristol-Myers Squibb. Dr Soverini acted as a consultant for and received honoraria from Novartis, Bristol-Myers Squibb, and ARIAD Pharmaceuticals, Inc. Dr Woodman is an employee and shareholder of Novartis. Dr Hochhaus acted as a consultant for and received honoraria and research funding from Novartis, Bristol-Myers Squibb, Pfizer Inc., and ARIAD Pharmaceuticals, Inc. Dr Kim acted as a consultant for and received honoraria from Novartis and Bristol-Myers Squibb. Dr Saglio acted as a consultant for and received honoraria from Novartis and Bristol-Myers Squibb. Dr Radich acted as a consultant for Novartis, Bristol-Myers Squibb, Pfizer Inc., and ARIAD Pharmaceuticals, Inc., and received research funding from Novartis.

## Authors’ contributions

AMS, AH, JPR, RCW, and TPH participated in the design of the study and analyzed and interpreted data. D-WK, EG, GM, GS, LB, MCM, SB, and SS collected, analyzed, and interpreted data. All authors drafted, read and approved the final manuscript.

## Pre-publication history

The pre-publication history for this paper can be accessed here:

http://www.biomedcentral.com/1471-2407/13/173/prepub
